# Collaborative efforts to forecast seasonal influenza in the United States, 2015–2016

**DOI:** 10.1038/s41598-018-36361-9

**Published:** 2019-01-24

**Authors:** Craig J. McGowan, Matthew Biggerstaff, Michael Johansson, Karyn M. Apfeldorf, Michal Ben-Nun, Logan Brooks, Matteo Convertino, Madhav Erraguntla, David C. Farrow, John Freeze, Saurav Ghosh, Sangwon Hyun, Sasikiran Kandula, Joceline Lega, Yang Liu, Nicholas Michaud, Haruka Morita, Jarad Niemi, Naren Ramakrishnan, Evan L. Ray, Nicholas G. Reich, Pete Riley, Jeffrey Shaman, Ryan Tibshirani, Alessandro Vespignani, Qian Zhang, Carrie Reed, The Influenza Forecasting Working Group, Roni Rosenfeld, Nehemias Ulloa, Katie Will, James Turtle, David Bacon, Steven Riley, Wan Yang

**Affiliations:** 1 Epidemiology and Prevention Branch, Influenza Division, Centers for Disease Control and Prevention, Atlanta, Georgia USA; 2 Dengue Branch, Division of Vector-Borne Diseases, Centers for Disease Control and Prevention, Atlanta, Georgia USA; 3 Arete Associates, Northridge, California, USA; 4 Predictive Science, Inc., San Diego, California, USA; 5 Computer Science Department, Carnegie Mellon University, Pittsburgh, Pennsylvania USA; 6 Division of Media and Network Technologies and Division of Frontier Science, Graduate School of Information Science and Technology, Gi-CoRE Station for Big Data & Cybersecurity, Hokkaido University, Sapporo, Japan; 7 Division of Environmental Health Sciences, School of Public Health, University of Minnesota, Minneapolis, Minnesota USA; 8 Knowledge Based Systems, Inc., College Station, Texas USA; 9 Computational Biology Department, Carnegie Mellon University, Pittsburgh, Pennsylvania USA; 10 Discovery Analytics Center, Virginia Tech University, Arlington, Virginia USA; 11 Department of Statistics and Data Science, Carnegie Mellon University, Pittsburgh, Pennsylvania USA; 12 Department of Environmental Health Sciences, Mailman School of Public Health, Columbia University, New York, New York, USA; 13 Department of Mathematics, University of Arizona, Tucson, Arizona USA; 14 Department of Statistics, University of California, Berkeley, Berkeley, California USA; 15 Department of Statistics, Iowa State University, Ames, Iowa USA; 16 Department of Mathematics and Statistics, Mount Holyoke College, South Hadley, Massachusetts USA; 17 Department of Biostatistics and Epidemiology, School of Public Health and Health Sciences, University of Massachusetts, Amherst, Amherst, Massachusetts USA; 18 Department of Statistics and Data Science, Machine Learning Department, Carnegie Mellon University, Pittsburgh, Pennsylvania USA; 19 Northeastern University, Boston, Massachusetts USA

## Abstract

Since 2013, the Centers for Disease Control and Prevention (CDC) has hosted an annual influenza season forecasting challenge. The 2015–2016 challenge consisted of weekly probabilistic forecasts of multiple targets, including fourteen models submitted by eleven teams. Forecast skill was evaluated using a modified logarithmic score. We averaged submitted forecasts into a mean ensemble model and compared them against predictions based on historical trends. Forecast skill was highest for seasonal peak intensity and short-term forecasts, while forecast skill for timing of season onset and peak week was generally low. Higher forecast skill was associated with team participation in previous influenza forecasting challenges and utilization of ensemble forecasting techniques. The mean ensemble consistently performed well and outperformed historical trend predictions. CDC and contributing teams will continue to advance influenza forecasting and work to improve the accuracy and reliability of forecasts to facilitate increased incorporation into public health response efforts.

## Introduction

Seasonal influenza epidemics result in substantial human health and financial burdens in the United States, with an estimated 140,000–710,000 hospitalizations and 12,000–56,000 deaths annually depending on the severity of the season^1,2^. The magnitude and timing of influenza epidemics vary from year to year^3,4^, making the annual impact difficult to predict. Current Centers for Disease Control and Prevention (CDC) surveillance systems track influenza activity nationwide in a variety of ways, including monitoring virologic characteristics, outpatient visits for influenza-like illness (ILI), hospitalizations, and mortality^5^. While these systems collect valuable data, they are intrinsically describing activity that occurred in the past and require data processing time, limiting their utility for real-time public health decision making. Accurate and timely forecasts of influenza activity could assist in the public health response to both seasonal epidemics and future pandemics.

Since the 2013–2014 influenza season, CDC has hosted collaborative challenges to forecast the timing, intensity, and short-term trajectory of ILI activity in the United States using data from the US Outpatient Influenza-like Illness Surveillance Network (ILINet), a robust and geographically broad surveillance system, as its benchmark^6,7^. While ILI can capture both influenza and non-influenza illnesses, it is of high public health value as it correlates strongly with laboratory confirmed influenza and its magnitude correlates well with other measures of seasonal influenza severity^8^.

To continue the advancement of forecasting science, the application of forecasts for public health decision-making, and the development of best practices, CDC and challenge participants update challenge guidelines each year. For example, after the first challenge, several improvements were made including standardizing forecast submission formats, requiring specification of probabilistic forecasts rather than point forecasts, and implementing fully quantitative forecast evaluation^9^. Additional changes were made for the 2015–2016 season to improve the public health utility of the forecasts. First, challenge participants provided forecasts with increased resolution for peak intensity and trajectory predictions, which allows for a more detailed interpretation of forecasts and flexibility in scoring forecast accuracy. In addition, the evaluation methodology was modified to allow for a pre-specified number of preceding and proceeding values to be considered correct to reduce the effect of revisions to ILINet on forecast scores. To help communicate forecasts in real-time, a public webpage to host predictions was created^10^.

In the present analysis, we summarized the results and insights gained from the 2015–2016 challenge and identified areas for improvement moving forwards. We also evaluated the performance of a simple average ensemble of the submitted influenza forecasts since ensemble forecasts have demonstrated several advantages over single forecast models in both weather and infectious disease forecasting^11–15^. Finally, we used gamma regression to investigate characteristics of both forecast models and influenza seasons that may be associated with increased forecast accuracy.

## Results

**Figure 1:**
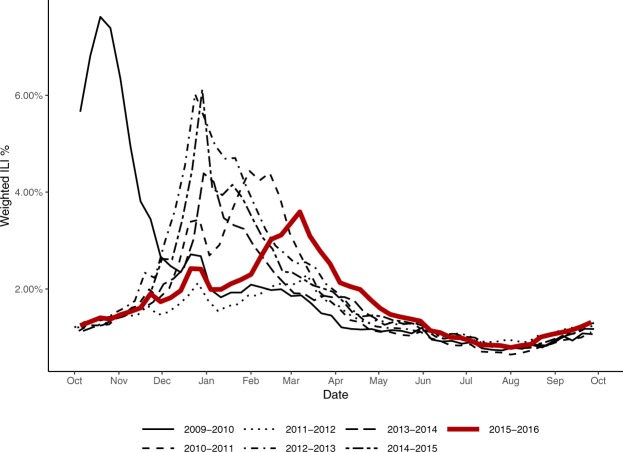
Percentage of visits for ILI reported by ILINet – 2009–2010 season to 2015–2016 season.

Figure 1 shows the national ILINet curve for the 2015–2016 season in comparison to the 2009–2010 through 2014–2015 seasons. Compared to earlier seasons, the 2015–2016 season started later and had a later peak. The peak intensity was 3.5%, well below the high value of 7.7% set in the 2009–2010 pandemic season and below the peak of 6.0% in 2014–2015. Seasonal forecast targets and evaluation periods for short–term forecasts for each region are shown in Table 1. The evaluation period for each target reflected the Morbidity and Mortality Weekly Report (MMWR) calendar weeks when forecasts for that target have the most utility (see Methods).

**Table 1:** 2015–2016 seasonal target values and boundaries of evaluation periods for the United States as a whole and each HHS Region, based on ILINet values published MMWR week 28 (July 22, 2016).

	Seasonal Targets				Evaluation Periods (MMWR Week)		
	Baseline value	Onset Week	Peak Week	Peak Intensity	Onset Week	Peak Week/ Intensity	1–4 week ahead
US National	2.1%	3	10	3.6%	42 − 9	42 − 14	51 − 17
HHS Region 1	1.3%	51	10	2.5%	42 − 5	42 − 17	47 − 18
HHS Region 2	2.3%	4	11	4.1%	42 − 10	42 − 14	52 − 17
HHS Region 3	1.8%	47	10	4.0%	42 − 1	42 − 18	43 − 18
HHS Region 4	1.6%	3	10	3.6%	42 − 9	42 − 18	51 − 18
HHS Region 5	1.6%	7	10	3.3%	42 − 13	42 − 14	3 − 17
HHS Region 6	3.6%	47	7	5.6%	42 − 1	42 − 13	49 − 16
HHS Region 7	1.7%	7	10	2.5%	42 − 13	42 − 14	3 − 17
HHS Region 8	1.4%	5	8, 11	2.2%	42 − 11	42 − 15	1 − 18
HHS Region 9	2.6%	3	7	4.4%	42 − 9	42 − 14	51 − 17
HHS Region 10	1.1%	2	7	2.4%	42 − 8	42 − 15	50 − 18

Eleven teams submitted fourteen separate forecasts (models A-N, Table 2). Table 2 contains brief descriptions of each model’s methodology. All model structures remained consistent over the season, and only Model G made minor updates to their method to better incorporate trends in ILINet revisions. All but one model provided predictions for each of the 10 HHS Regions. Most teams participated throughout the season, but four forecasts began late: Model I (MMWR week 50), Model K (MMWR week 45), Model L (MMWR week 49), and Model N (MMWR week 4). For these models, earlier forecasts were scored as missing.

**Table 2:** Participating model descriptions.

Model	Data source	Regional forecast^a^	Model type	Returning Team	Ensemble Forecast	Brief description
A	ILINet, weather attributes	Yes	Mechanistic^b^	Yes	Yes	SIRS model with ensemble Kalman filter to assimilate observed data sources.
B	ILINet	Yes	Statistical^c^	No	Yes	Historical predictions for part of season, followed by extra trees random forest predictive model.
C	ILINet, specific humidity	Yes	Mechanistic	Yes	Yes	SIR, SIRS, SEIR, SEIRS models combined using three ensemble filter algorithms w/ fixed scale and real-time ILI measures.^24–26^
D	ILINet, specific humidity	Yes	Mechanistic	Yes	Yes	SIR, SIRS, SEIR, SEIRS models combined using three ensemble filter algorithms w/ variable scale and inferred ILI measures^24–26^.
E	ILINet, Twitter, Wikipedia	Yes	Statistical	Yes	No	Kalman filter using archetypal ILI trajectory as a process model and digital surveillance as measurements^27^.
F	ILINet, crowd-sourced forecasts	Yes	Statistical	Yes	Yes	Aggregate forecast from many individual crowd-sourced forecasts^28^.
G	ILINet	Yes	Statistical	Yes	Yes	Weighted ensemble of ten statistical models including empirical Bayes, smooth splines, empirical distribution.
H	ILINet, weather attributes	Yes	Statistical	No	No	Use maximum mutual information to explore dependencies between factors and determine the optimal predictive model; variables included are ILI, temperature, rain/snowfall, leading to a maximum entropy generalized non-linear model.
I^d^	ILINet, Twitter	Yes	Statistical	No	No	Bayesian hierarchical model that borrows information from previous flu seasons to inform about the current flu season.
J	ILINet	Yes	Mechanistic	No	No	Fit optimal parabola to incidence curve for current season ILI data, incorporating noise estimated from past seasons^29^.
K^d^	ILINet	Yes	Statistical	No	No	Use k-nearest neighbours approach to select past season most similar to current season. Historical variance with normality assumption used to generate probabilities.
L^d^	ILINet	No	Statistical	No	No	Use kernel conditional density estimation to estimate each future week, combine using copulas to create joint distribution^30^.
M	ILINet, Twitter	Yes	Mechanistic	Yes	No	Uses Twitter and ILINet data to set initial conditions for stochastic generative epidemic model, calibrated to historical ILI surveillance^31,32^.
N^d^	ILINet, school vacation schedules, specific humidity	Yes	Mechanistic	No	No	An MCMC procedure with an SIR model using climate and school vacation schedule to determine the reproduction number. National forecasts are a weighted average of coupled regional forecasts.

^a^“Yes” denotes forecast for ≥1 HHS region (for all weeks).

^b^Includes models that incorporate compartmental modelling like Susceptible-Exposed-Infected-Recovered [SEIR] models.

^c^Includes models like time series analysis and generalized linear models.

^d^First forecast received on MMWR week 45 (Model K), 49 (Model L), 50 (Model I), and week 4 (Model N).

### Forecast Accuracy

Overall forecast accuracy was assessed using a metric of forecast skill, where 1 is a perfect forecast and 0 means the forecast assigned a <1% probability to the correct outcome. Average forecast skill for national targets over their respective evaluation periods are shown in Table 3. At the national level, median team forecast skill was highest for short-term forecasts of ILINet 1 week in advance (0.66) and decreased for the 2, 3, and 4 week ahead targets (median skill 0.41, 0.31, and 0.29, respectively). Median forecast skill for peak intensity (0.30) was comparable to that of short-term forecasts at 3–4 weeks. Median forecast skill for peak week and onset week are not directly comparable to the ILINet intensity forecasts because the scales and bins were different, but were both low (0.03 and 0.04, respectively).

**Table 3:** Average forecast skill for US national targets by forecast team during the 2015–2016 influenza season. **Bold** denotes the highest scoring team for that target.

	Onset week	Peak week	Peak intensity	Seasonal average^a^	1 week ahead	2 week ahead	3 week ahead	4 week ahead	Short-term average^b^
Model A	0.004	0.003	0.523	0.021	0.107	0.122	0.114	0.115	0.114
Model B	**0.179**	**0.204**	0.515	**0.274**	0.612	0.513	0.451	0.398	0.492
Model C	0.038	0.015	0.255	0.054	0.578	0.293	0.164	0.098	0.238
Model D	0.037	0.031	0.279	0.072	0.876	0.668	0.443	0.297	0.540
Model E	0.045	0.072	**0.655**	0.139	0.707	0.658	0.601	0.535	0.626
Model F	0.038	0.072	0.647	0.131	**0.893**	0.727	**0.663**	0.514	**0.695**
Model G	0.047	0.110	0.581	0.157	0.847	0.715	0.638	**0.577**	0.693
Model H	0.014	0.000	0.055	0.007	0.014	0.067	0.011	0.008	0.017
Model I^c^	0.004	0.008	0.013	0.008	0.162	0.209	0.257	0.317	0.225
Model J	0.000	0.155	0.383	0.036	0.711	0.399	0.303	0.207	0.376
Model K^c^	0.037	0.030	0.076	0.044	0.358	0.343	0.320	0.283	0.326
Model L^c^	0.105	0.167	0.323	0.185	0.747	**0.759**	0.566	0.352	0.590
Model M	0.004	0.021	0.278	0.033	0.698	0.426	0.284	0.169	0.357
Model N^c^	0.001	0.002	0.003	0.002	0.061	0.043	0.014	0.009	0.025
Median Team Skill	0.037	0.030	0.301	0.049	0.655	0.413	0.311	0.290	0.366
FluSight Ensemble	0.115	0.134	0.505	0.206	0.719	0.620	0.542	0.466	0.585
Hist. Avg. Forecast	0.108	0.054	0.268	0.117	0.406	0.408	0.404	0.400	0.404

^a^Average of submissions for onset week, peak week, and peak intensity.

^b^Average of submissions for 1, 2, 3, and 4 weeks ahead.

^c^First forecast received on MMWR week 45 (Model K), 49 (Model L), 50 (Model I), and week 4 (Model N); Missing forecasts are assigned a log score of −10 for scoring purposes.

At the national level, Model F had the highest skill for forecasts of ILINet 1 and 3 weeks ahead (0.89 and 0.66, respectively), as well as the highest average skill across all short-term targets (0.70). Model L had the highest skill for forecasts of ILINet 2 weeks ahead (0.76), while Model G had the highest skill for forecasts of ILINet 4 weeks ahead (0.58). Model B had the highest skill for both season onset and peak week (0.18 and 0.20, respectively), as well as the highest average skill across all seasonal targets (0.27). Model E had the highest skill for maximum intensity (0.66). Models E, F, G, and L had skills greater than the median team skill for all targets, while Model B had skills greater than the median team skill for all targets except forecasts of ILINet 1 week ahead.

**Figure 2:**
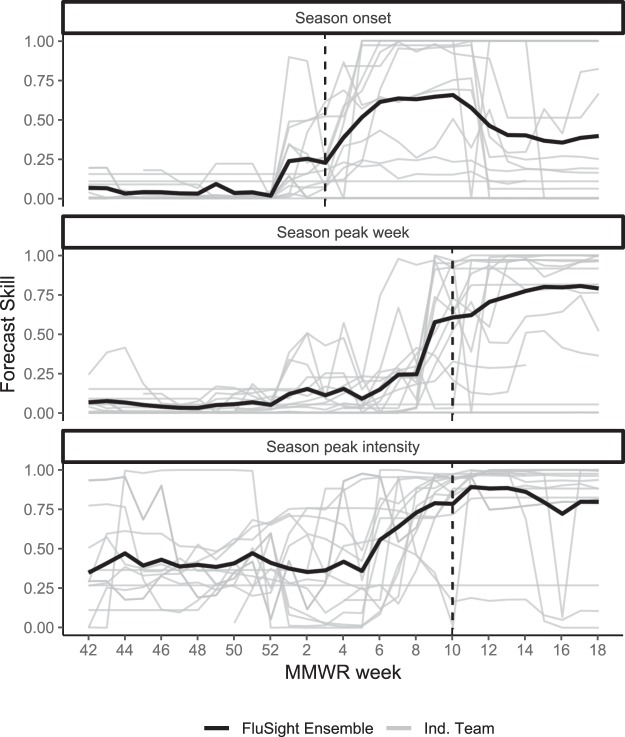
Weekly forecast skill for national onset week, season peak intensity, and season peak week during the 2015–2016 influenza season. Each grey line represents a separate forecast model, the solid black line represents the FluSight Ensemble, and vertical dashed lines indicate the date when the forecasted target occurred.

**Figure 3:**
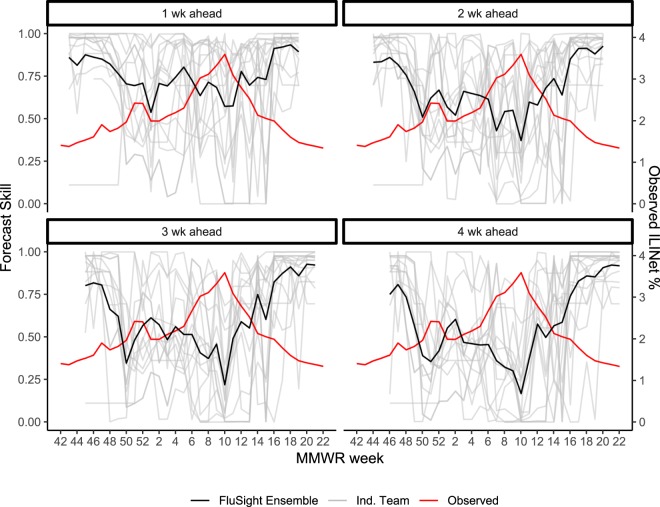
Weekly forecast skill for one to four week ahead forecasts of the national ILINet percentage for individual team forecasts shown in grey and for the FluSight Ensemble shown in black during the 2015–2016 season, by week, with the observed ILINet percent (wILI%) overlaid in red. The x-axis represents the MMWR week that each forecast is predicting.

As the season progressed, forecast skill for season onset, peak week, and peak intensity at the national level generally increased, though individual team skill varied considerably, especially for peak intensity (Fig. 2). For all seasonal targets, skill improved noticeably once the target of interest had passed according to observed ILINet data. For example, no models assigned a >50% probability to week 10 as the peak on week 6, while at week 10, 36% of submitted models assigned a >50% probability to week 10 as the peak. Week-ahead forecasts at the national level also showed considerable variability (Fig. 3), especially near the peak intensity of the influenza season when week-to-week variability in the ILINet value was the highest. All short-term forecasts had noticeable dips in accuracy around MMWR weeks 50 and 10, corresponding to inflection points in the ILINet data (Fig. 3).

At the regional level, median model forecast skill generally followed the same trend as the national level across the short-term forecasts, with 1 week ahead forecasts having the highest score and 4 week ahead forecasts having the lowest (Supplementary Tables S1–S10). Median forecast skill for peak intensity and onset week varied considerably across the regions, with scores ranging from 0.06 to 0.51 and 0.003 to 0.46, respectively. Median forecast skill for peak week was low across all regions, ranging from 0.006 to 0.15. Across regions, median model forecast skill for short-term targets was lowest in HHS Regions 6 and 9 and highest in HHS Regions 8 and 10, while median skill for season targets was highest in HHS Regions 9 and 10 and lowest in HHS Regions 2 and 4 (Table 4).

**Table 4:** Median team average forecast skill by target for forecast locations during the 2015-2016 influenza forecasting challenge. Bold denotes the location with the highest median team forecast skill.

Location	Onset week	Peak week	Peak intensity	1 week ahead	2 week ahead	3 week ahead	4 week ahead
US National	0.037	0.030	0.301	0.655	0.413	0.311	0.290
HHS Region 1	**0.456**	0.013	0.382	0.497	0.553	0.525	0.404
HHS Region 2	0.003	0.011	0.209	0.379	0.231	0.223	0.184
HHS Region 3	0.384	0.006	0.174	0.570	0.269	0.184	0.163
HHS Region 4	0.004	0.027	0.229	0.386	0.302	0.191	0.204
HHS Region 5	0.039	0.019	0.184	0.537	0.256	0.286	0.200
HHS Region 6	0.259	0.017	0.060	0.256	0.161	0.122	0.077
HHS Region 7	0.032	0.022	0.153	0.627	0.514	0.438	0.276
HHS Region 8	0.054	0.021	0.498	**0.735**	**0.677**	**0.582**	0.436
HHS Region 9	0.043	0.063	**0.514**	0.248	0.231	0.224	0.192
HHS Region 10	0.092	**0.137**	0.482	0.625	0.565	0.537	**0.492**

### FluSight Ensemble and Historical Average Comparisons

Nine models outperformed the model based on the historical average of ILINet data at the national level for both peak intensity and 1 week ahead forecasts, while seven models outperformed the historical average for 2 weeks ahead and six models outperformed the historical average for both peak week and 3 weeks ahead (Table 3). Only three models outperformed the historical average for forecasts of ILINet 4 weeks ahead and only one model outperformed the historical average for onset week. For all targets at the national level, a model consisting of the unweighted mean of submitted models, which we refer to as the FluSight Ensemble model, outperformed the majority of submitted models (Table 3). Similar performance was seen for forecast targets at the HHS Regional level (Supplementary Tables S1–S10).

### Gamma Regression by Model and Influenza Season Characteristics

**Figure 4:**
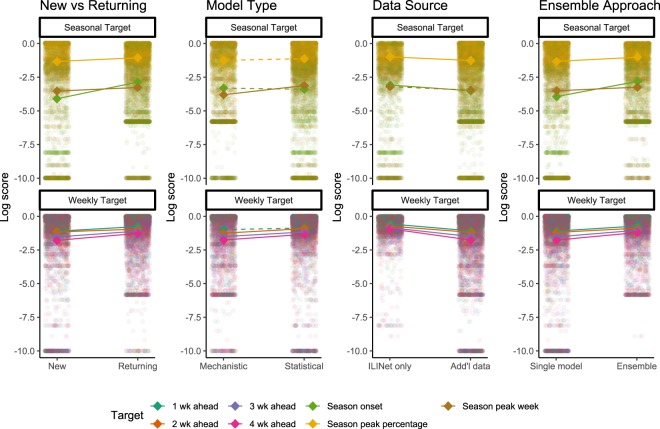
Log scores by characteristics of the forecasting approach. Each small, transparent point represents the log score for a specific target (colours), location, and forecast week. Seasonal targets are shown in the top panel and short-term targets in the bottom panel. Each sub-panel is divided by forecast characteristics including whether the team had participated in previous seasons, whether the model was mechanistic or statistical, whether data sources other than ILINet were used, and whether an ensemble was used to create the forecast. Bold diamonds represent the average log score across models for each target in each category. Solid lines indicate statistically significant differences determined by multivariable gamma regression controlling for location and forecast week.

Seven models were submitted by four teams that participated in the 2014–2015 CDC influenza forecasting challenges, though model specifications were updated between seasons. On average, these models were significantly more accurate than the seven forecasts submitted by first-time participating teams (Fig. 4). Five models utilized mechanistic models, encompassing compartmental modelling strategies such as Susceptible-Infected-Recovered (SIR) models. These methods attempted to model the underlying disease transmission dynamics and translated that into forecasts. Nine models utilized statistical methods that did not attempt to model disease transmission, but instead directly estimated the ILINet curve or target of interest using approaches such as time-series analysis or generalized linear models. The statistical models generally outperformed mechanistic models, with significant differences for peak week and 2, 3, and 4 week ahead forecasts. During the period between MMWR weeks 50-1, when there is a historic rise and dip in ILI values (Fig. 1), statistical models generally outperformed mechanistic models for 1–4 week ahead forecasts (Supplementary Table S11). Five models used only ILINet data to inform their predictions and nine models used additional data sources beyond those available in ILINet. The models using only ILINet data generally outperformed models incorporating additional data, with significant differences for all targets except peak week. Finally, six models used an ensemble approach combining predictions from multiple “in-house” models, and these models were associated with significantly higher skill for all forecasting targets compared to single models.

**Figure 5:**
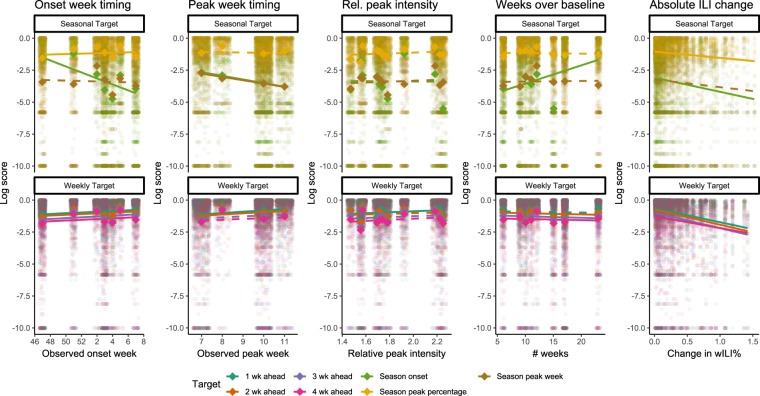
Log scores by characteristics of the influenza season. Each small, transparent point represents the log score for a specific target (colours), location, and forecast week. Seasonal targets are shown in the top panel and short-term targets in the bottom panel. Each sub-panel is divided by seasonal characteristics including observed timing of onset week, observed timing of peak week, relative intensity of the peak wILI% value to the baseline value, the number of weeks ILINet remained above baseline, and the absolute difference between the initial published wILI% value for the week a forecast is based on and the week’s final wILI% value. Bold diamonds represent the average log score across models for each target in each category. Solid lines indicate statistically significant differences determined by multivariable gamma regression controlling for forecast week.

We compared forecasts across all forecast locations (10 HHS regions and the entire United States) to assess how seasonal characteristics (timing of season onset and peak, level of peak intensity relative to baseline, number of weeks above baseline, revisions to initial published wILI% values) affected forecast skill for those targets. Forecasts of peak week and onset were less accurate and forecasts for 1 and 2 weeks ahead were slightly more accurate for locations with a later peak, though forecast skill for other targets was unaffected (Fig. 5). Similarly, forecasts of season onset were less accurate for locations with a later onset. Relative peak intensity, defined as the peak intensity for a location divided by that location’s baseline ILINet value, had a significant but small association with increased accuracy for forecasts of 1 week ahead, but was not associated with forecast accuracy for any other targets examined. Short-term forecasts were generally less accurate in locations with longer influenza seasons, as measured in the number of weeks wILI% was above baseline. Forecasts of season onset were significantly more accurate in locations with longer influenza seasons, while accuracy of peak week and peak intensity forecasts were not associated with the length of the influenza season. Forecasts of short-term targets were also less accurate for forecasts based on weeks with larger differences between the initial and final published ILINet values than weeks with smaller revisions.

### Comparison to 2014–2015 Forecasting Results

Both onset and peak week occurred much later in the 2015–2016 season compared to the 2014–2015 season; at the national level, onset occurred in week 47 in 2014–2015 and week 3 in 2015–2016, while peak week occurred in week 52 in 2014–2015 and week 10 in 2015–2016. Compared to scores from the 2014–2015 challenge^9^, median team skill was higher for each of the national 1–4 week ahead targets in the 2015–2016 challenge than in the 2014–2015 challenge when scored using the same metrics (Table 5). The top model skill for each short-term target also increased from 2014–2015 to 2015–2016. The median skill for national peak percentage was higher during the 2015–2016 challenge, while the top model skill remained the same. However, median and top model skill for national onset week and peak week were both lower during the 2015–2016 challenge compared to the 2014–2015 challenge.

**Table 5:** Median and top team forecast skill for national targets from 2014–2015 and 2015–2016 influenza challenge, using scoring rules from 2014–2015 challenge^a^.

	Onset week	Peak week	Peak intensity	Seasonal average^b^	1 week ahead	2 week ahead	3 week ahead	4 week ahead	Short-term average^c^
2015/2016 Median Team Skill	0.01	0.02	0.22	0.02	0.34	0.23	0.19	0.16	0.23
2014/2015 Median Team Skill	0.04	0.25	0.02	0.11	0.14	0.11	0.08	0.10	0.13
2015/2016 Top Team Skill	0.04	0.06	0.35	0.07	0.63	0.58	0.45	0.39	0.46
2014/2015 Top Team Skill	0.41	0.49	0.35	0.39	0.43	0.30	0.34	0.34	0.34

^a^For 2014-2015, forecasts for peak intensity and short-term forecasts were binned as semi-open 1% bins up to 10%, with a final bin for all values greater than or equal to 10%. For all targets, only the probability assigned to the correct bin was considered correct for scoring^9^.

^b^Average of submissions for onset week, peak week, and peak intensity.

^c^Average of submissions for 1, 2, 3, and 4 weeks ahead.

## Discussion

The 2015–2016 influenza season was the third consecutive influenza season that CDC hosted an influenza forecasting challenge. This accumulating body of real-time forecast data provides new insights on forecast accuracy, relative model performance, the value of ensemble approaches, and the challenges of influenza forecasting.

Forecast skill varied as the season progressed. Short-term forecast skill was generally highest at during the shoulders of the season when ILINet values were low and relatively constant and lowest around the peak week, an inflection point of the ILINet curve and a period during the influenza season when forecasts likely have the highest value from a public health perspective (Fig. 3). Forecast accuracy for seasonal targets, on the other hand, generally improved throughout the season as models incorporated new data. Skill for the seasonal targets generally began to improve substantially between 2 and 4 weeks prior to the predicted event (Fig. 2). While this improved accuracy may in part reflect more accurate short-term forecasts, the identification of the change from increasing to decreasing incidence (i.e. the peak) is a critical milestone for decision-makers. Even a lead time of only a few weeks is helpful for situational awareness, especially with a reporting delay of 1 to 2 weeks for initial surveillance data, subsequent revisions to those data as reporting is completed, and week-to-week variation that may occur even in the complete surveillance data. These forecasts can therefore provide public health officials with some level of confidence that the event has occurred.

A comparison of forecast skill across the forecast locations revealed additional characteristics of forecast performance. Forecasts for onset week and peak week generally had lower skill in locations with later onset weeks and peak weeks (Fig. 5). Seasonal targets that occur particularly early or late in a flu season are likely harder to predict simply because they are atypical, possibly with respect to other locations in the same season, with respect to previous seasons in the same location, or both. For short-term forecasts, these effects were not as strong (Fig. 5), indicating that late seasons have less of an effect on short-term forecasts. Conversely, short-term forecasts based on weeks with large subsequent revisions to the originally published ILINet values were less accurate than forecasts based on weeks that had minimal revisions to the final ILINet values. This is supported by the low median scores for short-term targets seen in HHS Regions 6 and 9 (Table 4), both of which had among the highest levels of backfill during the 2015–2016 season (Supplementary Fig. S1).

Compared to the previous season (2014–2015), average forecast skill in 2015–2016 was higher for peak intensity and lower for onset week and peak week (Table 5). The higher skill for peak intensity may reflect that 2014–2015 was an abnormally intense season while the peak intensity for 2015–2016 was more in line with typical past seasons (Fig. 1). Meanwhile, the onset and peak occurred later than typical in 2015–2016, possibly leading to the lower forecast skill for onset week and peak week. Notably, this agrees with our finding that forecast skill was lower for locations with later onset and peak weeks. The short-term forecasts had higher average forecast skill in 2015–2016 compared to 2014–2015. This may reflect short-term dynamics that were easier to predict, but more likely indicates higher model accuracy as this improvement was seen across locations where dynamics were quite different (Table 5).

Overall, there was no single best model across all targets; eleven of the fourteen participating models had the highest average score for at least one of the 77 short-term and seasonal targets across the 10 HHS regions and the United States. Nonetheless, Model B and Models E, F, G, and L consistently outperformed the FluSight Ensemble, other models, and the historical average for the seasonal and short-term targets at the national level, respectively. Also of note, the FluSight Ensemble outperformed the majority of individual forecast models for all targets and the historical average for all seven targets at the national level, showing that the combined forecasts provided more reliable information than most specific forecasts and more information than historical data alone. As the FluSight Ensemble was a simple average of received forecasts, the application of more sophisticated ensemble methods offers an opportunity for further improvements. The intention of the FluSight Ensemble was to evaluate a simple *a priori* ensemble approach that could be used during the season to combine information from multiple models, and as such we did not evaluate *a posteriori* approaches that could not be applied in real-time.

The variation in accuracy between models and the wide variety of forecasting approaches also provides insight into the characteristics of more accurate models. Comparisons of these approaches are not generalizable because they only reflect the combination of characteristics included in the submitted models, nothing close to the full spectrum of possible approaches^16^. They nonetheless provide notable insights. Models submitted by teams who had competed in the CDC forecasting challenge in previous years generally outperformed models submitted by new teams (Fig. 4). This may reflect self-selection of high-performing teams deciding to continue participating or it may indicate the value of participating in previous challenges. Making and submitting updated probabilistic forecasts on a weekly basis is a substantial technical challenge and those with experience doing that may be in a better position to identify and implement changes to improve accuracy. Models using ensemble approaches to generate their forecasts also outperformed single models, providing additional evidence of the value of ensemble forecasting approaches. Models that used data in addition to ILINet were less accurate than those only using ILINet data for six of seven targets, indicating that including auxiliary data does not necessarily lead to more accurate forecasts. Comparisons between statistical and mechanistic approaches indicated that performance varied by target, with statistical models outperforming mechanistic models for four of seven targets. However, the five models that consistently outperformed the median skill for all targets were all statistical models, illustrating the potential for this forecasting method. Additionally, during MMWR weeks 50 – 1, when there historically is a peak and dip in ILI values (Fig. 1), statistical models outperformed mechanistic models (Supplementary Table S11), illustrating that the statistical approaches may be more resilient to predictable patterns in ILI. As more forecasting approaches are applied over more seasons, more locations, and more diseases, more substantive analyses of these differences will be possible.

As an open, standardized, real-time, forecasting challenge, the CDC influenza forecasting challenge has provided unique insight into epidemic forecasting. The results highlight the continuing challenge of improving forecast accuracy for more seasons and at lead times of several weeks or more, forecasts that would be of even more utility for public health officials. To improve future forecasts, we found evidence that experience may help, that there is room for improving the use of external data, and that combining forecasts from multiple models in ensembles improved accuracy. Despite remaining challenges, both the top models and the FluSight Ensemble provided more accurate forecasts than historical data alone. Moreover, the accuracy for more typical seasons and for nearer targets (e.g. 1-week vs. 4-week ahead forecasts or peak forecasts early in the season vs. as the peak approaches) indicates that the models are producing valuable information as is. Because these forecasts are available in real time, they can actively improve situational awareness and be used to directly address immediate public health needs such as planning for hospital staffing and bed availability, outbreak preparedness, and stocking of antivirals.

Interest in infectious disease forecasting has increased in recent years, with challenges to predict epidemics of both chikungunya^17^ and dengue fever^18^ in addition to influenza. As the only ongoing infectious disease forecasting challenge in the United States, the CDC influenza forecasting challenge sets a model for other infectious diseases by identifying data and resource constraints that limit model development, establishing best practices for forecast submission and evaluation, identifying areas where forecasts can be improved, tying forecasting efforts to real public health needs, and assessing their performance related to those needs.

## Methods

### Challenge Structure

Teams from the previous challenge as well as research groups with experience in influenza or infectious disease forecasting worked with CDC to define the structure for the 2015–2016 challenge. Teams submitted weekly forecasts from November 2, 2015, to May 16, 2016. Forecasting targets were based on data from ILINet, a syndromic surveillance system consisting of more than 2,000 outpatient providers^5^. These providers send CDC weekly reports consisting of the number of patients with ILI and the total number of patients seen. These reports are weighted based on state population to determine a weighted percentage of patient visits due to ILI (wILI%). ILINet data use the Morbidity and Mortality Weekly Report (MMWR) week system, where a week starts on a Sunday and ends on a Saturday. Data for a given MMWR week are usually released the following Friday in CDC’s weekly FluView publication^19^. Each week’s publication includes initial values for the most recent week as well as potential revisions of prior published values, and the difference between initial and final published value varies by week and region (Supplementary Fig. S1).

Forecasting targets included seasonal and short-term targets. To participate in the challenge, teams were required to submit predictions on each target for the United States as a whole and had an option to submit predictions for each of the ten HHS Regions. The seasonal targets were onset week, defined as the first week where wILI% was at or above the location-specific baseline and remained at or above for at least two additional weeks; peak week, defined as the MMWR week during which the wILI% was highest; and peak intensity, defined as the season-wide maximum wILI%. Short-term targets included forecasts of wILI% one-, two-, three-, and four-weeks in advance of FluView publication. Due to the delay in reporting surveillance data (e.g. data for MMWR week 50 is published on MMWR week 51), the short-term targets provide a forecast for ILINet activity that occurred in the past week (1-week ahead), the present week (2-weeks ahead), and 1 (3-weeks ahead) and 2 weeks (4-weeks ahead) in the future.

As in the 2014–2015 season, participants submitted forecasts weekly as point estimates and probability distributions in a series of bins categorized across possible values for each target. For onset week and peak week, there was a bin for each single week of the season, with an additional bin for onset week corresponding to no onset. For peak intensity and short-term forecasts, semi-open 0.5% intervals (e.g. 1.0% ≤ wILI% < 1.5%) were used from 0% up to 13%, with the final bin representing the probability for all values greater than or equal to 13%. Teams submitted a written narrative of their forecasting methods for each model. Changes to the methods and narrative description were permitted during the season.

This study did not involve human participants and institutional review board approval was not required.

### Historical Average Forecast

To provide a benchmark to compare submitted forecasts to, we created a historical average forecast using ILINet data from the 1997–1998 flu season through the 2014–2015 flu season, excluding the 2009–2010 H1N1 pandemic year as its dynamics were atypical compared to seasonal epidemics. A Gaussian kernel density estimate using bandwidths estimated by the Sheather-Jones method^20^ was fitted to each MMWR week’s previous observed ILINet values, and approximate probabilities for each prediction bin were calculated by integrating the kernel density^9^. The point estimate was the median of the estimated distribution. Forecasts for onset week, peak week and peak intensity were calculated in the same way. Onset week forecast probabilities were adjusted to reflect the probability of no onset week based on the percentage of prior years in which ILI values did not cross the region-specific baseline. As CDC only began publishing regional baselines with the 2007–08 flu season, only seasons from that point onwards were used to calculate the onset week forecasts.

### Unweighted FluSight Ensemble

To evaluate the utility of a simple ensemble of influenza forecasts, we constructed an unweighted average of the individual forecasts received, which we refer to as the FluSight Ensemble. The estimated distribution of the FluSight Ensemble was created by taking the arithmetic mean of all submitted distributions for a given target/location combination during a particular week. As with the historical average forecast, we used the median of each distribution as the point estimate.

### Forecast Evaluation

We compared the forecasts, including the historical average and FluSight Ensemble forecasts, to weighted ILINet data published on MMWR week 28 (ending July 16, 2016), which was chosen *a priori* to represent final ILINet values for the season. We scored the forecasts using a forecast skill metric derived from the logarithmic scoring rule^21,22^. Let ***p*** be the binned probabilities submitted for a given forecast target, with *p*_*i*_ the probability assigned to the bin containing the observed outcome *i*. For all targets, we included the bin above (*i* + 1) and below (*i* – 1) the observed outcome, and calculated the logarithmic score as $S({\boldsymbol{p}},\,{i})={\rm{ln}}({p}_{i-1}+{p}_{i}+{p}_{i+1}).$ For example, if the peak week was MMWR week 10, the logarithmic score would be calculated by summing the probabilities assigned to MMWR weeks 9–11 and taking the natural logarithm of that sum. In the case of multiple weeks having the same maximum wILI% and therefore being peak weeks, both peak weeks were considered as observed outcomes and the bins surrounding each peak were also included in the calculated score. Scores below −10, missing forecasts, or forecasts that summed to probabilities less than 0.9 or greater than 1.1 were all assigned scores of −10. Scores were averaged across different combinations of locations, targets, and time periods. As in 2014–2015, the averaged log scores were exponentiated to create a forecast skill on a scale of 0 to 1. Perfect forecasts would receive a skill of 1, while forecasts that assign very low probabilities to the observed outcome would receive a skill close to 0.

Evaluation periods varied by target and were chosen at the end of the season to include the weeks in which forecasts would have had the most utility to public health decision makers. The evaluation period for each seasonal target began with the first forecast submitted (MMWR week 42) and ended a target-specific number of weeks after each outcome had occurred. For onset week, the evaluation period ended six weeks after the season onset. For peak week and peak intensity, the evaluation period extended until one week after wILI% went below baseline and stayed below baseline for the remainder of the season (Table 1). For short-term forecasts, the evaluation period for each location began with forecasts received four weeks prior to season onset in that location and extended to 4 weeks after ILINet returned below baseline for that location.

We utilized gamma regression to analyse the effect of model type, data sources, targets, absolute change between initial and final published wILI% in the week each forecast was based on, and season types (e.g., late vs. early defined continuously by season onset and peak week) on forecast accuracy characterized as the negative log score. Gamma regression is restricted to outcome values greater than or equal to zero and is well-suited for analysing right-skewed data. For all regression models, we analysed across all weekly forecasts, targets and locations, excluding week-target-location forecasts that were not submitted. For comparisons of model characteristics, we controlled for location and the week a forecast was received in the regression analysis. For comparisons of seasonal characteristics across regions, we controlled for the week a forecast was received.

To compare forecasts across seasons, we summarized the 2015–2016 forecasts received into the larger, 1% wide bins utilized in the 2014–2015 challenge and scored the forecasts using the 2014–2015 log scoring rules. Forecasts for onset week and peak week were scored the same way during the 2014–2015 season, while for peak intensity and the short-term targets, only the probability assigned to the bin containing the observed value ${p}_{i}$ was used.

Analyses were conducted using R version 3.4.3^23^ and significance was assessed using a cutoff of p < 0.05.

## Electronic supplementary material

Supplementary Information

## Data Availability

The received forecasts that support the findings of this study are publicly available on the CDC Epidemic Prediction Initiative GitHub page at https://github.com/cdcepi/FluSight-forecasts.
