# Supplementary material for: Collaborative efforts to forecast seasonal influenza in the United States, 2015–2016
Source: Sci Rep. 2019 Jan 24;9:683. doi: 10.1038/s41598-018-36361-9 (PMC6346105; doi:10.1038/s41598-018-36361-9)

Supplemental Material

Collaborative efforts to forecast seasonal influenza in the United States, 2015–2016

Craig J McGowan[1], Matthew Biggerstaff*[1], Michael Johansson[2], Karyn M Apfeldorf[3], Michal Ben-Nun[4], Logan Brooks[5], Matteo Convertino[6, 7], Madhav Erraguntla[8], David C Farrow[9], John Freeze[8], Saurav Ghosh[10], Sangwon Hyun[11], Sasikiran Kandula[12], Joceline Lega[13], Yang Liu[7], Nicholas Michaud[14], Haruka Morita[12], Jarad Niemi[15], Naren Ramakrishnan[10], Evan L Ray[16], Nicholas G Reich[17], Pete Riley[4], Jeffrey Shaman[12], Ryan Tibshirani[18], Alessandro Vespignani[19], Qian Zhang[19], Carrie Reed[1], and the Influenza Forecasting Working Group

[1]Epidemiology and Prevention Branch, Influenza Division; [2]Dengue Branch, Division of Vector-Borne Diseases, Centers for Disease Control and Prevention, Atlanta, Georgia, USA.

[3]Arete Associates, Northridge, California, USA

[4]Predictive Science, Inc., San Diego, California, USA

[5]Computer Science Department, Carnegie Mellon University, Pittsburgh, Pennsylvania, USA

[6]Division of Media and Network Technologies and Division of Frontier Science, Graduate School of Information Science and Technology, Gi-CoRE Station for Big Data & Cybersecurity, Hokkaido University, Sapporo, Japan

[7]Division of Environmental Health Sciences, School of Public Health, University of Minnesota, Minneapolis, Minnesota, USA

[8]Knowledge Based Systems, Inc., College Station, Texas, USA

[9]Computational Biology Department, Carnegie Mellon University, Pittsburgh, Pennsylvania, USA

[10]Discovery Analytics Center, Virginia Tech University, Arlington, Virginia, USA

[11]Department of Statistics and Data Science, Carnegie Mellon University, Pittsburgh, Pennsylvania, USA

[12]Department of Environmental Health Sciences, Mailman School of Public Health, Columbia University, New York, New York, USA

[13]Department of Mathematics, University of Arizona, Tucson, Arizona, USA

[14]Department of Statistics, University of California, Berkeley, Berkeley, California, USA

[15]Department of Statistics, Iowa State University, Ames, Iowa, USA

[16]Department of Mathematics and Statistics, Mount Holyoke College, South Hadley, Massachusetts, USA

[17]Department of Biostatistics and Epidemiology, School of Public Health and Health Sciences, University of Massachusetts, Amherst, Amherst, Massachusetts, USA

[18]Department of Statistics and Data Science, Machine Learning Department, Carnegie Mellon University, Pittsburgh, Pennsylvania, USA

[19]Northeastern University, Boston, Massachusetts, USA.

Influenza Forecasting Working Group:

Roni Rosenfeld[20], Nehemias Ulloa[21], Katie Will[21], James Turtle[22] David Bacon[23], Steven Riley[22], and Wan Yang[24]

[20] Computer Science Department, Carnegie Mellon University, Pittsburgh, Pennsylvania, USA

[21] Department of Statistics, Iowa State University, Ames, Iowa, USA

[22] Predictive Science, Inc., San Diego, California, USA

[23] Leidos, Reston, Virginia, USA

[24] Department of Environmental Health Sciences, Mailman School of Public Health, Columbia University, New York, New York, USA

Supplementary Table S1: Average forecast skill for HHS Region 1 targets by forecast team during the 2015-2016 influenza season. **Bold** denotes the highest scoring team for that target.

	Onset week	Peak week	Peak intensity	Seasonal average[a]	1 week ahead	2 week ahead	3 week ahead	4 week ahead	Short-term average[b]
Model A	0.595	0.010	0.382	0.102	0.260	0.348	0.314	0.292	0.302
Model B	0.266	0.066	0.616	0.215	0.733	0.673	0.701	**0.668**	0.694
Model C	0.533	0.025	0.271	0.126	0.497	0.370	0.257	0.202	0.316
Model D	0.504	0.044	0.350	0.171	0.832	0.717	0.566	0.404	0.614
Model E	0.456	0.118	0.577	0.297	0.596	0.553	0.525	0.472	0.536
Model F	**0.639**	**0.125**	0.700	**0.353**	0.689	0.648	0.633	0.557	0.631
Model G	0.468	0.017	0.505	0.135	0.731	0.661	0.638	0.594	0.656
Model H	0.475	0.001	0.074	0.018	0.037	0.103	0.033	0.013	0.036
Model I[c]	0.000	0.009	0.016	0.004	0.042	0.041	0.054	0.071	0.050
Model J	0.385	0.013	**0.739**	0.136	**0.882**	**0.782**	0.664	0.608	**0.730**
Model K[c]	0.024	0.052	0.080	0.052	0.396	0.356	0.329	0.291	0.342
Model L[c]	0.259	0.010	0.440	0.092	0.416	0.717	**0.702**	0.650	0.606
Model M	0.000	0.002	0.004	0.001	0.015	0.010	0.008	0.005	0.009
Median Team Skill	0.456	0.017	0.382	0.126	0.497	0.553	0.525	0.404	0.536
Hist. Avg. Forecast	0.207	0.116	0.310	0.193	0.469	0.470	0.458	0.444	0.461
FluSight Ensemble	0.491	0.131	0.513	0.300	0.654	0.611	0.578	0.539	0.595

[a] Average of submissions for onset week, peak week, and peak intensity

[b] Average of submissions for 1, 2, 3, and 4 weeks ahead

[c] First forecast received on MMWR week 45 (Model K), 49 (Model L), 50 (Model I), and week 4 (Model N)

Supplementary Table S2: Average forecast skill for HHS Region 2 targets by forecast team during the 2015-2016 influenza season. **Bold** denotes the highest scoring team for that target.

	Onset week	Peak week	Peak intensity	Seasonal average[a]	1 week ahead	2 week ahead	3 week ahead	4 week ahead	Short-term average[b]
Model A	0.003	0.003	0.149	0.012	0.132	0.172	0.137	0.101	0.134
Model B	0.002	0.003	0.432	0.015	**0.544**	**0.530**	0.481	0.425	0.496
Model C	0.040	0.015	0.271	0.056	0.459	0.516	**0.555**	**0.538**	**0.513**
Model D	0.035	0.027	0.209	0.060	0.223	0.231	0.223	0.184	0.215
Model E	**0.059**	**0.141**	**0.523**	**0.171**	0.403	0.433	0.396	0.392	0.406
Model F	0.036	0.033	0.434	0.083	0.419	0.479	0.399	0.361	0.414
Model G	0.036	0.049	0.499	0.100	0.529	0.512	0.440	0.424	0.477
Model H	0.001	0.000	0.073	0.004	0.379	0.226	0.076	0.040	0.135
Model I[c]	0.002	0.014	0.017	0.009	0.081	0.126	0.164	0.169	0.127
Model J	0.003	0.001	0.456	0.009	0.393	0.362	0.342	0.331	0.358
Model K[c]	0.002	0.011	0.015	0.007	0.187	0.161	0.145	0.160	0.163
Model L[c]	0.005	0.006	0.080	0.013	0.038	0.045	0.032	0.029	0.036
Model M	0.000	0.001	0.001	0.000	0.023	0.016	0.003	0.002	0.008
Median Team Skill	0.003	0.011	0.209	0.013	0.379	0.231	0.223	0.184	0.215
Hist. Avg. Forecast	0.152	0.016	0.282	0.086	0.341	0.334	0.323	0.317	0.329
FluSight Ensemble	0.072	0.096	0.398	0.144	0.452	0.445	0.398	0.374	0.418

[a] Average of submissions for onset week, peak week, and peak intensity

[b] Average of submissions for 1, 2, 3, and 4 weeks ahead

[c] First forecast received on MMWR week 45 (Model K), 49 (Model L), 50 (Model I), and week 4 (Model N)

Supplementary Table S3: Average forecast skill for HHS Region 3 targets by forecast team during the 2015-2016 influenza season. **Bold** denotes the highest scoring team for that target.

	Onset week	Peak week	Peak intensity	Seasonal average[a]	1 week ahead	2 week ahead	3 week ahead	4 week ahead	Short-term average[b]
Model A	0.330	0.003	0.632	0.062	0.162	0.159	0.135	0.086	0.131
Model B	0.566	0.003	0.431	0.057	0.576	0.427	0.355	0.302	0.403
Model C	0.384	0.029	0.152	0.090	0.448	0.253	0.184	0.140	0.233
Model D	0.436	0.025	0.147	0.085	0.676	0.425	0.312	0.224	0.377
Model E	0.320	**0.348**	**0.659**	**0.447**	0.645	0.559	0.461	0.402	0.509
Model F	**0.694**	0.229	0.489	0.380	**0.772**	**0.672**	0.551	0.503	**0.616**
Model G	0.446	0.236	0.394	0.326	0.716	0.601	0.526	0.482	0.575
Model H	0.064	0.003	0.051	0.016	0.570	0.269	0.145	0.099	0.217
Model I[c]	0.001	0.002	0.007	0.003	0.012	0.010	0.009	0.008	0.010
Model J	0.643	0.003	0.235	0.046	0.656	0.595	**0.580**	**0.563**	0.598
Model K[c]	0.004	0.048	0.093	0.041	0.168	0.161	0.163	0.163	0.164
Model L[c]	0.509	0.006	0.174	0.050	0.440	0.193	0.125	0.107	0.184
Model M	0.000	0.005	0.006	0.002	0.007	0.005	0.003	0.003	0.005
Median Team Skill	0.384	0.006	0.174	0.057	0.570	0.269	0.184	0.163	0.233
Hist. Avg. Forecast	0.116	0.119	0.184	0.142	0.422	0.425	0.425	0.421	0.423
FluSight Ensemble	0.444	0.161	0.419	0.285	0.623	0.531	0.472	0.432	0.509

[a] Average of submissions for onset week, peak week, and peak intensity

[b] Average of submissions for 1, 2, 3, and 4 weeks ahead

[c] First forecast received on MMWR week 45 (Model K), 49 (Model L), 50 (Model I), and week 4 (Model N)

Supplementary Table S4: Average forecast skill for HHS Region 4 targets by forecast team during the 2015-2016 influenza season. **Bold** denotes the highest scoring team for that target.

	Onset week	Peak week	Peak intensity	Seasonal average[a]	1 week ahead	2 week ahead	3 week ahead	4 week ahead	Short-term average[b]
Model A	0.003	0.003	0.229	0.015	0.014	0.022	0.026	0.030	0.022
Model B	0.002	0.005	0.436	0.019	0.474	0.400	0.352	0.339	0.388
Model C	0.032	0.033	0.241	0.069	0.511	0.302	0.191	0.123	0.245
Model D	0.019	0.045	0.219	0.065	0.698	0.545	0.367	0.233	0.425
Model E	0.013	0.114	**0.648**	0.124	0.615	0.627	**0.637**	**0.590**	**0.617**
Model F	0.004	**0.151**	0.578	0.100	0.740	0.627	0.474	0.360	0.530
Model G	0.026	0.143	0.505	**0.148**	**0.777**	**0.658**	0.554	0.475	0.606
Model H	0.000	0.002	0.039	0.003	0.048	0.142	0.037	0.018	0.046
Model I[c]	0.004	0.004	0.005	0.004	0.089	0.074	0.052	0.038	0.060
Model J	0.000	0.027	0.148	0.016	0.277	0.135	0.075	0.071	0.119
Model K[c]	0.001	0.053	0.047	0.020	0.386	0.392	0.363	0.329	0.367
Model L[c]	0.004	0.018	0.405	0.038	0.171	0.094	0.128	0.204	0.143
Model M	0.000	0.004	0.006	0.002	0.058	0.022	0.021	0.010	0.023
Median Team Skill	0.004	0.027	0.229	0.020	0.386	0.302	0.191	0.204	0.245
Hist. Avg. Forecast	**0.194**	0.002	0.309	0.045	0.251	0.258	0.267	0.272	0.262
FluSight Ensemble	0.052	0.132	0.405	0.158	0.560	0.489	0.437	0.399	0.468

[a] Average of submissions for onset week, peak week, and peak intensity

[b] Average of submissions for 1, 2, 3, and 4 weeks ahead

[c] First forecast received on MMWR week 45 (Model K), 49 (Model L), 50 (Model I), and week 4 (Model N)

Supplementary Table S5: Average forecast skill for HHS Region 5 targets by forecast team during the 2015-2016 influenza season. **Bold** denotes the highest scoring team for that target.

	Onset week	Peak week	Peak intensity	Seasonal average[a]	1 week ahead	2 week ahead	3 week ahead	4 week ahead	Short-term average[b]
Model A	0.003	0.004	0.150	0.013	0.129	0.097	0.130	0.175	0.129
Model B	0.087	**0.218**	0.562	**0.220**	0.771	0.702	0.665	0.614	0.691
Model C	0.066	0.019	0.184	0.061	0.537	0.219	0.113	0.049	0.173
Model D	0.043	0.028	0.180	0.060	0.817	0.539	0.356	0.200	0.441
Model E	0.089	0.155	**0.607**	0.204	0.724	0.691	0.659	**0.678**	0.689
Model F	0.046	0.061	0.576	0.117	**0.914**	**0.800**	**0.694**	0.666	**0.771**
Model G	**0.144**	0.011	0.520	0.094	0.899	0.750	0.649	0.606	0.727
Model H	0.002	0.001	0.066	0.006	0.080	0.038	0.057	0.026	0.047
Model I[c]	0.002	0.008	0.013	0.006	0.126	0.183	0.286	0.194	0.186
Model J	0.000	0.184	0.214	0.025	0.289	0.210	0.248	0.324	0.263
Model K[c]	0.039	0.037	0.073	0.047	0.287	0.256	0.230	0.197	0.243
Model L[c]	0.010	0.003	0.294	0.020	0.879	0.275	0.343	0.283	0.404
Model M	0.001	0.002	0.002	0.001	0.388	0.188	0.064	0.017	0.105
Median Team Skill	0.039	0.019	0.184	0.047	0.537	0.256	0.286	0.200	0.263
Hist. Avg. Forecast	0.127	0.140	0.388	0.191	0.425	0.439	0.446	0.453	0.440
FluSight Ensemble	0.092	0.163	0.423	0.185	0.670	0.564	0.513	0.466	0.555

[a] Average of submissions for onset week, peak week, and peak intensity

[b] Average of submissions for 1, 2, 3, and 4 weeks ahead

[c] First forecast received on MMWR week 45 (Model K), 49 (Model L), 50 (Model I), and week 4 (Model N)

Supplementary Table S6: Average forecast skill for HHS Region 6 targets by forecast team during the 2015-2016 influenza season. **Bold** denotes the highest scoring team for that target.

	Onset week	Peak week	Peak intensity	Seasonal average[a]	1 week ahead	2 week ahead	3 week ahead	4 week ahead	Short-term average[b]
Model A	**0.713**	0.004	0.041	0.028	0.086	0.089	0.080	0.068	0.080
Model B	0.259	0.086	0.339	0.187	0.326	0.288	0.240	0.193	0.259
Model C	0.451	0.013	0.384	0.104	0.256	0.161	0.122	0.102	0.153
Model D	0.472	0.016	**0.505**	0.127	0.551	0.338	0.188	0.188	0.290
Model E	0.540	0.187	0.047	0.135	0.358	0.201	0.125	0.068	0.162
Model F	0.516	0.206	0.243	0.266	**0.588**	**0.500**	**0.419**	**0.337**	**0.456**
Model G	0.188	**0.361**	0.299	**0.293**	0.505	0.404	0.334	0.302	0.382
Model H	0.006	0.019	0.008	0.010	0.001	0.008	0.002	0.001	0.002
Model I[c]	0.000	0.006	0.025	0.004	0.026	0.032	0.025	0.021	0.026
Model J	0.090	0.017	0.060	0.040	0.395	0.266	0.202	0.132	0.235
Model K[c]	0.012	0.059	0.046	0.038	0.104	0.097	0.089	0.077	0.092
Model L[c]	0.537	0.012	0.175	0.078	0.157	0.067	0.050	0.042	0.070
Model M	0.000	0.001	0.002	0.001	0.002	0.002	0.001	0.001	0.001
Median Team Skill	0.259	0.017	0.060	0.078	0.256	0.161	0.122	0.077	0.153
Hist. Avg. Forecast	0.222	0.226	0.137	0.184	0.223	0.225	0.227	0.223	0.224
FluSight Ensemble	0.438	0.169	0.292	0.256	0.432	0.360	0.304	0.265	0.338

[a] Average of submissions for onset week, peak week, and peak intensity

[b] Average of submissions for 1, 2, 3, and 4 weeks ahead

[c] First forecast received on MMWR week 45 (Model K), 49 (Model L), 50 (Model I), and week 4 (Model N)

Supplementary Table S7: Average forecast skill for HHS Region 7 targets by forecast team during the 2015-2016 influenza season. **Bold** denotes the highest scoring team for that target.

	Onset week	Peak week	Peak intensity	Seasonal average[a]	1 week ahead	2 week ahead	3 week ahead	4 week ahead	Short-term average[b]
Model A	0.003	0.003	0.037	0.007	0.078	0.095	0.191	0.225	0.128
Model B	0.059	0.190	0.261	0.143	0.799	0.778	0.682	**0.620**	0.722
Model C	0.053	0.024	0.153	0.058	0.653	0.332	0.158	0.099	0.257
Model D	0.032	0.022	0.172	0.049	0.888	0.725	0.438	0.276	0.550
Model E	**0.095**	**0.194**	0.254	**0.168**	0.612	0.514	0.442	0.403	0.494
Model F	0.052	0.045	0.305	0.089	0.819	0.740	0.704	0.604	0.719
Model G	0.088	0.014	**0.422**	0.081	0.796	0.633	0.565	0.517	0.628
Model H	0.000	0.000	0.061	0.002	0.036	0.100	0.014	0.004	0.023
Model I[c]	0.001	0.002	0.026	0.004	0.107	0.165	0.217	0.177	0.159
Model J	0.001	0.048	0.198	0.023	0.627	0.581	0.573	0.555	0.586
Model K[c]	0.043	0.031	0.063	0.044	0.304	0.269	0.234	0.186	0.248
Model L[c]	0.012	0.004	0.143	0.019	**0.966**	**0.889**	**0.894**	0.552	**0.821**
Model M	0.001	0.001	0.002	0.001	0.343	0.233	0.076	0.028	0.125
Median Team Skill	0.032	0.022	0.153	0.044	0.627	0.514	0.438	0.276	0.494
Hist. Avg. Forecast	0.055	0.096	0.168	0.096	0.376	0.390	0.417	0.436	0.402
FluSight Ensemble	0.086	0.128	0.332	0.154	0.664	0.584	0.511	0.451	0.554

[a] Average of submissions for onset week, peak week, and peak intensity

[b] Average of submissions for 1, 2, 3, and 4 weeks ahead

[c] First forecast received on MMWR week 45 (Model K), 49 (Model L), 50 (Model I), and week 4 (Model N)

Supplementary Table S8: Average forecast skill for HHS Region 8 targets by forecast team during the 2015-2016 influenza season. **Bold** denotes the highest scoring team for that target.

	Onset week	Peak week	Peak intensity	Seasonal average[a]	1 week ahead	2 week ahead	3 week ahead	4 week ahead	Short-term average[b]
Model A	0.003	0.009	0.298	0.023	0.307	0.323	0.332	0.373	0.331
Model B	0.254	**0.517**	0.490	0.408	0.735	0.707	0.727	0.729	0.725
Model C	0.136	0.088	0.511	0.185	0.835	0.624	0.437	0.335	0.539
Model D	0.111	0.087	0.498	0.172	0.913	0.806	0.680	0.436	0.697
Model E	0.276	0.426	0.609	**0.423**	0.722	0.677	0.645	0.631	0.670
Model F	0.122	0.292	0.664	0.297	0.945	0.891	0.840	**0.808**	0.873
Model G	**0.313**	0.002	0.656	0.071	0.894	0.799	0.724	0.682	0.776
Model H	0.003	0.015	0.664	0.034	0.266	0.200	0.053	0.037	0.107
Model I[c]	0.000	0.021	0.030	0.006	0.138	0.197	0.286	0.435	0.234
Model J	0.047	0.000	**0.701**	0.022	0.787	0.680	0.582	0.546	0.649
Model K[c]	0.054	0.018	0.081	0.043	0.397	0.370	0.336	0.297	0.351
Model L[c]	0.031	0.024	0.389	0.069	**0.991**	**0.941**	**0.884**	0.799	**0.906**
Model M	0.001	0.003	0.004	0.002	0.160	0.111	0.098	0.073	0.108
Median Team Skill	0.054	0.021	0.498	0.069	0.735	0.677	0.582	0.436	0.649
Hist. Avg. Forecast	0.225	0.263	0.277	0.256	0.526	0.529	0.536	0.539	0.532
FluSight Ensemble	0.185	0.333	0.564	0.334	0.763	0.703	0.651	0.602	0.682

[a] Average of submissions for onset week, peak week, and peak intensity

[b] Average of submissions for 1, 2, 3, and 4 weeks ahead

[c] First forecast received on MMWR week 45 (Model K), 49 (Model L), 50 (Model I), and week 4 (Model N)

Supplementary Table S9: Average forecast skill for HHS Region 9 targets by forecast team during the 2015-2016 influenza season. **Bold** denotes the highest scoring team for that target.

	Onset week	Peak week	Peak intensity	Seasonal average[a]	1 week ahead	2 week ahead	3 week ahead	4 week ahead	Short-term average[b]
Model A	0.003	0.003	**0.916**	0.024	0.233	0.204	0.264	0.269	0.240
Model B	0.061	0.392	0.730	0.282	0.582	0.431	0.436	0.519	0.489
Model C	**0.199**	0.253	0.204	0.218	0.271	0.239	0.190	0.153	0.211
Model D	0.154	0.113	0.233	0.159	0.365	0.291	0.253	0.160	0.261
Model E	0.182	**0.416**	0.603	**0.372**	0.605	0.548	0.531	0.527	0.554
Model F	0.085	0.105	0.632	0.186	0.464	0.414	0.478	0.480	0.457
Model G	0.099	0.240	0.559	0.249	**0.641**	**0.559**	**0.552**	**0.531**	**0.572**
Model H	0.002	0.001	0.046	0.005	0.007	0.027	0.013	0.004	0.010
Model I[c]	0.002	0.014	0.013	0.008	0.107	0.161	0.172	0.155	0.145
Model J	0.043	0.001	0.514	0.027	0.210	0.091	0.064	0.072	0.099
Model K[c]	0.038	0.063	0.050	0.050	0.248	0.231	0.212	0.192	0.221
Model L[c]	0.027	0.011	0.534	0.056	0.187	0.148	0.224	0.234	0.194
Model M	0.000	0.002	0.002	0.001	0.028	0.014	0.007	0.004	0.011
Median Team Skill	0.043	0.063	0.514	0.056	0.248	0.231	0.224	0.192	0.221
Hist. Avg. Forecast	0.301	0.171	0.272	0.238	0.378	0.392	0.405	0.414	0.396
FluSight Ensemble	0.166	0.256	0.522	0.290	0.505	0.470	0.440	0.422	0.460

[a] Average of submissions for onset week, peak week, and peak intensity

[b] Average of submissions for 1, 2, 3, and 4 weeks ahead

[c] First forecast received on MMWR week 45 (Model K), 49 (Model L), 50 (Model I), and week 4 (Model N)

Supplementary Table S10: Average forecast skill for HHS Region 10 targets by forecast team during the 2015-2016 influenza season. **Bold** denotes the highest scoring team for that target.

	Onset week	Peak week	Peak intensity	Seasonal average[a]	1 week ahead	2 week ahead	3 week ahead	4 week ahead	Short-term average[b]
Model A	0.004	0.020	0.680	0.046	0.331	0.301	0.392	0.396	0.351
Model B	0.122	**0.478**	0.490	**0.331**	0.636	0.565	0.587	0.538	0.582
Model C	**0.202**	0.366	0.291	0.286	0.852	0.762	0.713	0.609	0.734
Model D	0.196	0.295	0.283	0.260	0.829	0.744	0.688	0.492	0.684
Model E	0.092	0.392	0.482	0.283	0.625	0.587	0.580	0.552	0.587
Model F	0.106	0.392	0.520	0.303	0.833	0.756	**0.750**	0.749	0.773
Model G	0.167	0.195	0.395	0.241	0.656	0.565	0.537	0.521	0.570
Model H	0.005	0.152	**0.722**	0.105	0.070	0.114	0.085	0.033	0.070
Model I[c]	0.000	0.014	0.030	0.007	0.154	0.212	0.308	0.465	0.255
Model J	0.101	0.000	0.523	0.024	0.340	0.245	0.197	0.262	0.258
Model K[c]	0.041	0.066	0.085	0.063	0.308	0.295	0.288	0.275	0.292
Model L[c]	0.035	0.038	0.581	0.100	**0.952**	**0.819**	0.738	**0.791**	**0.825**
Model M	0.001	0.003	0.004	0.002	0.047	0.040	0.031	0.025	0.035
Median Team Skill	0.092	0.152	0.482	0.105	0.625	0.565	0.537	0.492	0.570
Hist. Avg. Forecast	0.160	0.195	0.101	0.145	0.375	0.380	0.397	0.410	0.389
FluSight Ensemble	0.184	0.326	0.526	0.331	0.679	0.625	0.603	0.568	0.620

[a] Average of submissions for onset week, peak week, and peak intensity

[b] Average of submissions for 1, 2, 3, and 4 weeks ahead

[c] First forecast received on MMWR week 45 (Model K), 49 (Model L), 50 (Model I), and week 4 (Model N)

Supplementary Table S11: Median average team score for mechanistic models and statistical models across all regions for targets during MMWR weeks 50, 51, 52, and 1, when there is a historical upwards and downwards trend in wILI% in many regions.

Target	Mechanistic Model	Statistical Model
1 week ahead	-2.569	-0.899
2 week ahead	-2.793	-0.870
3 week ahead	-2.821	-0.846
4 week ahead	-2.860	-0.932
Onset week	-4.101	-3.745
Peak week	-4.906	-3.821
Peak intensity	-2.608	-1.144

Supplementary Figure S1: Difference between initial published value of wILI% and final published value of wILI% by MMWR week throughout the 2015-2016 influenza season. Positive values indicate the final value was higher than the initial value.

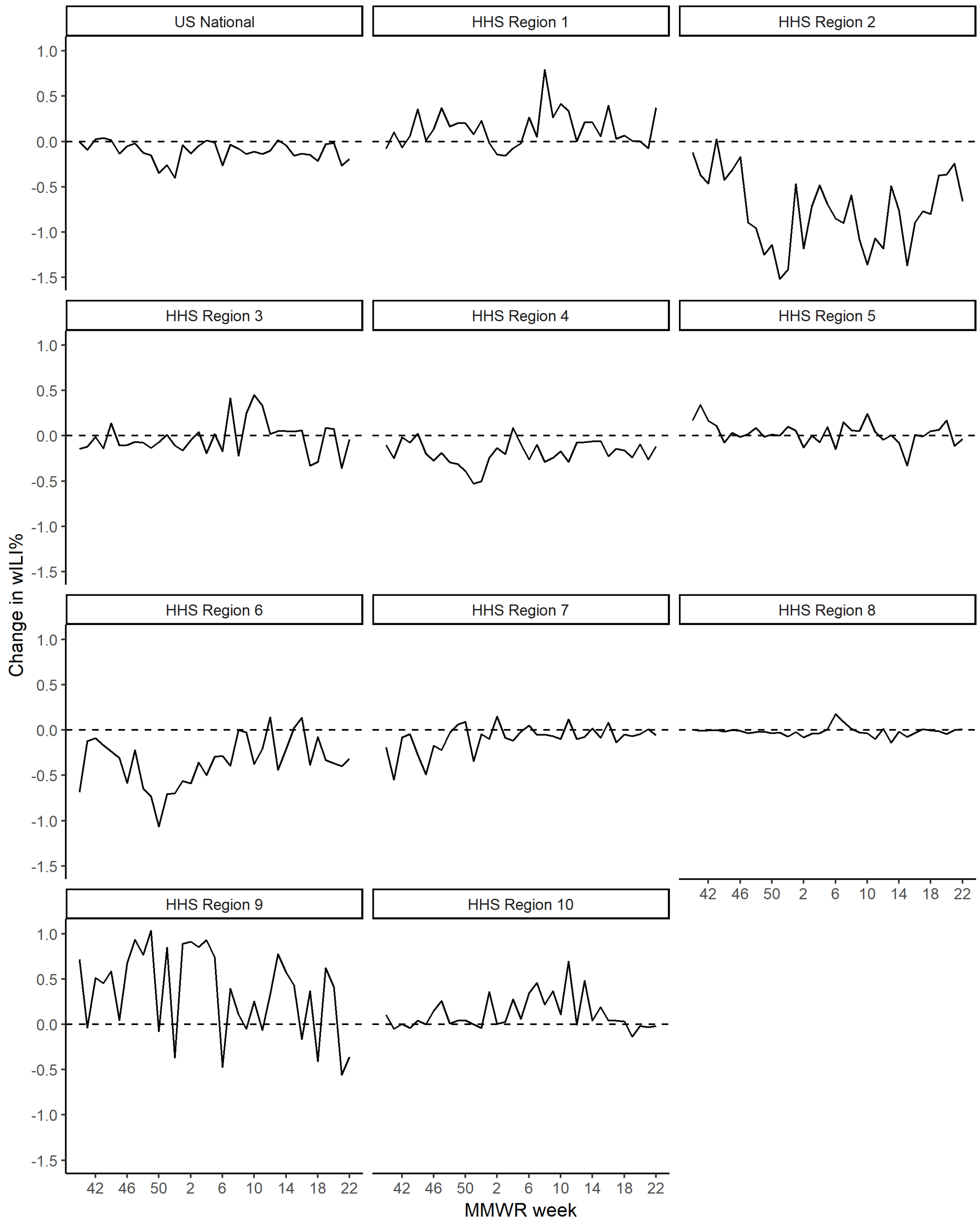

Supplement: Supplementary file 1 — Supplementary Information [file 41598_2018_36361_MOESM1_ESM.pdf]
